# The awareness-distress paradox: Traffic-related air pollution awareness and psychological distress among the parents of urban schoolchildren in a northeastern province, Thailand

**DOI:** 10.1371/journal.pone.0358849

**Published:** 2026-09-21

**Authors:** Rajitra Nawawonganun, Kittipong Sornlorm, Roshan Kumar Mahato

**Affiliations:** Faculty of Public Health, Khon Kaen University, Khon Kaen, Thailand; Universidad Santiago de Cali, COLOMBIA

## Abstract

Urban air pollution is increasingly recognized as a mental-health risk, yet its impact on parents of schoolchildren remains understudied, particularly in rapidly urbanizing Southeast Asian cities. This study examined the prevalence of, and factors associated with, psychological distress related to traffic-related air pollution (TRAP) awareness and exposure among parents of urban schoolchildren in Khon Kaen Municipality, a major urban center in northeastern Thailand. A cross-sectional study surveyed 557 parents from two primary schools in high-traffic areas during March–May 2025, Thailand’s hot/dry high-pollution season. Structured interviews assessed sociodemographic characteristics, air pollution awareness and exposure, health behaviors, physical symptoms, and psychological distress using a validated 10-item questionnaire. Data were analyzed by bivariate and multivariable logistic regression with backward elimination (α = 0.05). Moderate-to-high psychological distress affected 61.94% of parents (95% confidence interval [CI]: 57.87–65.85). Higher air pollution awareness showed the strongest association with distress, following a graded pattern (moderate: adjusted odds ratio [AOR] = 2.41, 95%CI: 1.56–3.72; high: AOR = 4.39, 95%CI: 2.33–8.30). Other significant factors were moderate-to-appropriate health behaviors (AOR = 3.36, 95%CI: 2.14–5.26), air quality monitoring app use (AOR = 1.79, 95%CI: 1.17–2.74), and four to five affected body systems (AOR = 2.16, 95%CI: 1.32–3.53). A cross-sectional awareness–distress pattern was identified, whereby greater air pollution awareness was associated with higher psychological distress; the design does not permit conclusions about direction of effect. School-based air-quality communication in traffic-exposed urban settings should pair risk information with practical protective guidance and mental-health support for caregivers, relevant to rapidly urbanizing cities across Southeast Asia and beyond.

## Introduction

Air pollution represents one of the most pressing global environmental health challenges, contributing to an estimated 6.7 million premature deaths annually [1]. While the adverse effects of air pollution on the respiratory and cardiovascular systems have been extensively documented, a growing body of evidence indicates that air pollution exposure also significantly affects mental health outcomes, including mood disorders, anxiety symptoms, and broader forms of psychological distress [2,3]. A recent umbrella review synthesizing 32 meta-analyses found suggestive to highly suggestive evidence linking air pollution exposure to mood disorders, anxiety, and suicidal behavior, establishing air pollution as a modifiable risk factor for adverse mental health outcomes [3].

Urban environments, where traffic-related air pollution (TRAP) is concentrated, present particular challenges for mental health. TRAP is a complex mixture of fine particulate matter, nitrogen oxides, carbon monoxide, and volatile organic compounds emitted from vehicle exhaust and road activity. Beyond well-established respiratory and cardiovascular effects, exposure to TRAP and fine particulate matter has been linked to neuroinflammation, oxidative stress, sleep disturbance, and anxiety-, depression-, and stress-related symptoms. In Southeast Asia, rapid urbanization and increasing motorization have led to deteriorating air quality in major urban centers [4]. Thailand, with substantial regional urbanization and rapid growth in road transport, experiences pronounced seasonal episodes of particulate matter pollution, with PM_2.5_ concentrations frequently exceeding World Health Organization guidelines, especially during the hot and dry season from roughly December through April [5]. During these periods, public health advisories and extensive media coverage of hazardous air quality levels become pervasive, potentially amplifying risk perception and environmental awareness among urban populations. Such heightened awareness may also add to caregivers’ psychological burden.

Parents of schoolchildren represent a particularly vulnerable group for air pollution-related psychological distress, as they bear the dual burden of concern for their own health and that of their children. Research has demonstrated that parental worry about environmental threats can significantly impact psychological well-being, a phenomenon that overlaps conceptually with eco-anxiety, although it may manifest more broadly as psychological distress [6,7]. A recent systematic review found consistent positive correlations between eco-anxiety and symptoms of psychological distress, depression, and anxiety [8]. Furthermore, studies have shown that environmental awareness, while essential for promoting protective behaviors, may paradoxically intensify psychological distress when perceived environmental threats are viewed as chronic and uncontrollable [9,10].

Despite growing recognition of the air pollution-mental health nexus, significant research gaps remain. Most studies have focused on direct exposure pathways, examining clinical mental health outcomes in relation to measured pollutant concentrations [11,12]. However, the psychological mechanisms through which air pollution affects mental health extend beyond direct toxicological effects to include awareness-mediated pathways, where knowledge of environmental hazards may contribute to heightened psychological distress through affective responses such as worry and anxiety-related symptoms [13,14]. This awareness-mediated pathway is particularly relevant in contexts where air pollution is highly visible and extensively reported in the media, potentially intensifying psychological distress among parents responsible for safeguarding their children’s health during pollution seasons.

Furthermore, caregiver-focused evidence on air pollution and mental health from Thailand and other Southeast Asian urban settings remains limited, despite the region’s high exposure burden. A companion study from the same research group recently documented a high prevalence of respiratory symptoms (87.6%) among schoolchildren in the same study population, highlighting the significant health burden of urban air pollution in this setting [15]. While that study focused on children’s physical health outcomes, the mental health impact on their parents, who are responsible for managing exposure risks and making daily decisions about their children’s commute and activities, has not been examined.

This study aimed to determine the prevalence of psychological distress related to traffic-related air pollution exposure among parents of urban schoolchildren and to examine awareness-mediated and behavioral factors associated with this distress. In particular, this study investigated whether higher levels of air pollution awareness were associated with increased psychological distress, reflecting a potential awareness–distress paradox. The findings are intended to inform the development of integrated public health interventions that address both the physical and psychological dimensions of air pollution exposure in urban communities.

## Methods

### Study design and setting

This cross-sectional analytical study was conducted from January to May 2025 using face-to-face interviews with parents of selected students. The overall study period included coordination with relevant authorities and preparatory procedures prior to data collection. Data collection was undertaken from 1 March to 31 May 2025, following ethical approval, in Khon Kaen Municipality, a major urban center in northeastern Thailand with approximately 101,398 residents [16]. Khon Kaen serves as a regional economic hub with high volumes of private cars and motorcycles, particularly during morning (07:00–08:30) and afternoon (15:00–17:00) rush hours. The data-collection period corresponds to Thailand’s hot/dry season, a period of elevated regional particulate pollution associated with agricultural and open biomass burning, extending into the early transition to the southwest monsoon around mid-May. The questionnaire’s physical-symptom items used a six-month recall window; therefore, reported symptoms may reflect exposure accumulated across preceding cool/dry and haze-season conditions in addition to conditions at interview. Previous studies have documented ambient PM_2.5_ concentrations that frequently exceed WHO air quality guidelines in this setting, particularly during the hot and dry season [4]. Seven schools in the municipality met the eligibility criterion of being located directly adjacent to, or within a few hundred metres of, major arterial roads with signalised intersections and frequent congestion, and therefore of entailing substantial exposure to traffic-related air pollution. Two of these schools (Anuban Khon Kaen School and Suan Sanuk School) were randomly selected for the study.

### Study population and sampling

The study population comprised parents or guardians of students enrolled in the selected schools. Inclusion criteria required participants to commute with their child during rush hours, have at least six months of commuting experience, and provide informed consent. Parents who were seriously ill or unable to complete the interview were excluded. A sample size of 557 was calculated using the formula for multiple logistic regression [17], based on parameters from a prior study on PM2.5 exposure and health outcomes [5]. The following parameters were applied: P0 = 0.37, P1 = 0.54, B = 0.40, α = 0.05, β = 0.20, ρ = 0.50, and a variance inflation factor of 2.00, derived from prior PM_2.5_ related respiratory health evidence, yielding an adjusted minimum of 556.48, rounded to 557. A multi-stage random sampling method was used. The sampling frame comprised the seven eligible kindergarten-to-primary schools described above, all of which were located in high-traffic areas of Khon Kaen Municipality. In the first stage, two schools were randomly selected from this frame using computer-generated random numbers, and the calculated sample size was allocated proportional to total student enrolment (322 and 235 participants). In the second stage, the sampling frame within each school was stratified into kindergarten and primary levels with proportional allocation. In the third stage, individual students were selected using computer-generated random numbers, and their parents or guardians were invited for interview. All 557 selected participants completed the study, yielding a 100% response rate.

### Data collection instrument

Face-to-face structured interviews lasting approximately 30 minutes were conducted by trained research assistants. Prior to fieldwork, all research assistants underwent standardized training led by the principal investigator and co-investigators, covering study objectives, informed consent and child assent procedures, neutral item-by-item administration, privacy protection, and completeness checks after each interview, to ensure consistency across interviewers. The questionnaire was developed by the research team based on the study conceptual framework and relevant literature, drawing on the Health Belief Model for risk perception and preventive behavior and the Transactional Model of Stress and Coping for the mental-health and coping items. It comprised five sections: (1) sociodemographic characteristics, including gender, age, BMI, education, occupation, healthcare coverage, smoking status, alcohol consumption, and chronic disease history; (2) air pollution awareness and exposure including a 5-item awareness subscale measuring concern about local air quality, knowledge of health effects, attention to air quality reports, personal protective behavior, and community-level concern; (3) health behaviors covering exercise, nutrition, and rest/sleep practices; (4) physical symptoms potentially related to air pollution, organized by body system: respiratory, cardiovascular, neurological, dermatological, and immune/general; and (5) psychological distress, assessing anxiety, worry, stress, frustration, and helplessness related to air pollution.

Content validity was assessed by five subject-matter experts, yielding an Item-Objective Congruence (IOC) index of 0.75. Reliability was evaluated by administering the revised questionnaire to 30 parents from a school with similar environmental conditions; Cronbach’s alpha greater than 0.70 was set as the criterion for acceptable reliability, and the pilot yielded an overall Cronbach’s alpha of 0.82. Because several sections comprised factual characteristics, exposure checklists, or symptom checklists across distinct domains rather than reflective items measuring a single latent construct, internal consistency was reported for the overall multi-item instrument. In response to peer review, scale-specific psychometric properties were additionally examined in the full analytic sample for the three multi-item scales used in the regression models (air pollution awareness, health behaviour, and psychological distress), comprising Cronbach’s alpha for each scale, inter-scale correlations, exploratory factor analysis of the awareness and distress items, and formal assessment of discriminant validity using the Fornell–Larcker criterion and the heterotrait–monotrait (HTMT) ratio (S1 Appendix).

### Outcome variable

The primary outcome was psychological distress related to air pollution, measured using a 10-item questionnaire. Each item was scored on a 5-point Likert scale (1 = never, 2 = sometimes, 3 = frequently, 4 = very often, 5 = always), yielding a total score ranging from 10 to 50. Based on the theoretical score distribution and standard criterion-referenced cut-points commonly applied in Thai health-behavior research, scores were categorized as low (<23 points), moderate (23–36 points), or high (≥37 points) distress. For logistic regression analysis, the outcome was dichotomized as moderate-to-high distress (≥23 points) versus low distress (<23 points).

### Independent variables

Independent variables were organized into four domains: (1) personal characteristics, including gender (male/female), age (<36, 36–45, ≥ 46 years), BMI classified by Asian criteria (underweight <18.5, normal 18.5–22.9, overweight 23.0–24.9, obese ≥25.0 kg/m^2^) following Asian BMI criteria, education level, healthcare coverage, smoking status, alcohol consumption, and chronic disease; (2) awareness and exposure factors, including air pollution awareness score categorized as low (<10), moderate (10–13), or high (≥14) from the 5-item subscale (range 5–20), air quality monitoring application use, face mask wearing behavior, and close smokers in the household; (3) health behaviors, categorized by total score from 18 items as inappropriate (<42), moderate (42–65), or appropriate (≥66); following peer review, and because the appropriate category contained few participants (n = 40) and yielded an imprecise and unstable estimate, health behaviour was collapsed into two levels (inappropriate versus moderate-to-appropriate) for the regression analyses, while the three-level distribution is retained in Table 1 and both specifications are compared in S1 Appendix; and (4) physical symptoms, including parent respiratory symptoms and number of body systems affected (0–1, 2–3, 4–5 systems out of five systems assessed).

**Table 1:** Baseline characteristics of the study population (n = 557).

Characteristics	Number	Percentage
**Personal characteristics**		
**Gender**		
Male	77	13.82
Female	480	86.18
**Family status**		
Parents (Father/Mother)	507	91.02
Relatives/Caregivers	50	8.98
**Age (years)**		
<36	127	22.80
36-45	315	56.55
≥46	115	20.65
Mean (SD)	40.77 (7.44)	
Median (Min:Max)	40.00 (19:72)	
**BMI (kg/m²)**		
Underweight (<18.5)	31	5.57
Normal (18.5–22.9)	210	37.70
Overweight (23.0–24.9)	108	19.39
Obese (≥25.0)	208	37.34
Mean (SD)	24.41 (4.42)	
Median (Min:Max)	23.62 (14.69:42.24)	
**Education level**		
Below bachelor’s degree	85	15.26
Bachelor’s degree or diploma	407	73.07
Above bachelor’s degree	65	11.67
**Healthcare coverage**		
Universal Health Coverage	173	31.06
Private Insurance/Social Security	237	42.55
Civil Servant Scheme	147	26.39
**Smoking status**		
Non-smoker	534	95.87
Former smoker	16	2.87
Current smoker	7	1.26
**Alcohol consumption**		
Non-drinker	408	73.25
Former drinker	46	8.26
Current drinker	103	18.49
**Chronic disease**		
None	453	81.33
Present	104	18.67
**Awareness and exposure factors**		
**Air pollution awareness level (F1-F5 score)**		
Low (<10 scores)	140	25.13
Moderate (10–13 scores)	324	58.17
High (≥14 scores)	93	16.70
Mean (SD)	11.06 (2.63)	
Median (Min:Max)	11.00 (5:20)	
**Close smokers in household**		
None	323	58.00
Present	234	42.00
**Air quality monitoring app use**		
No	392	70.38
Yes	165	29.62
**Face mask wearing during pollution**		
No	350	62.84
Yes	207	37.16
**PM**_2.5_ knowledge at school zone		
Not aware	460	82.58
Aware	97	17.42
**Health behaviors**		
**Overall health behavior score (G1-G18)**		
Inappropriate (<42 scores)	118	21.18
Moderate (42–65 scores)	399	71.64
Appropriate (≥66 scores)	40	7.18
Mean (SD)	49.85 (10.39)	
Median (Min:Max)	49.00 (29:90)	
**Physical symptoms**		
**Parent respiratory symptoms (H1-H4)**		
No symptoms	137	24.60
Present (≥1 symptom)	420	75.40
**Number of body systems affected**		
0-1 systems	110	19.75
2-3 systems	172	30.88
4-5 systems	275	49.37

Note: SD, standard deviation; Min, minimum; Max, maximum; BMI, body mass index.

### Statistical analysis

Descriptive statistics were used to summarize participant characteristics as frequencies and percentages for categorical variables and means with standard deviations for continuous variables. The prevalence of psychological distress and individual symptom items were reported with 95% confidence intervals. Bivariate logistic regression was conducted to examine the association between each independent variable and psychological distress, with crude odds ratios (COR) and 95% confidence intervals. Variables with p ≤ 0.25 in bivariate analysis were eligible for inclusion in the multivariable model, following established methodological recommendations [18]. Potential confounders were addressed through multivariable logistic regression: all variables meeting the p ≤ 0.25 screening criterion, together with conceptually important variables, were entered simultaneously, and a backward elimination procedure was used to obtain the final parsimonious model while adjusting for the remaining covariates. Adjusted odds ratios (AOR) with 95% confidence intervals were reported, and statistical significance was set at α = 0.05. Model adequacy was assessed using the Hosmer-Lemeshow goodness-of-fit test and discriminative ability by the area under the receiver operating characteristic curve (AUC). Because the awareness and distress instruments both refer to air pollution, the distinguishability of the two constructs was examined before modelling: Cronbach’s alpha was computed separately for each scale, an exploratory factor analysis with promax rotation was fitted to the combined awareness and distress items, and discriminant validity was assessed using the Fornell–Larcker criterion and the HTMT ratio. Robustness of the final model was examined through prespecified sensitivity analyses using the continuous distress score, alternative distress cut-points, an a priori adjustment set instead of backward elimination, collinearity diagnostics (variance inflation factors), and influence diagnostics (S1 Appendix). All analyses were performed using Stata version 18.0 (StataCorp LLC, College Station, TX, USA) and R version 4.3.

### Ethical considerations

This study was approved by the Human Research Ethics Committee of Khon Kaen University (Approval No. HE672303; approval date: 24 February 2025) and conducted in accordance with the Declaration of Helsinki. Written informed consent was obtained from all participants.

## Results

### Baseline characteristics

Table 1 presents the baseline characteristics of the 557 study participants. The majority were female (86.18%), aged 36–45 years (56.55%), and parents of the enrolled children (91.02%). The mean age was 40.77 years (SD = 7.44; median = 40.00; range 19–72). Regarding BMI, 37.34% were classified as obese (≥25.0 kg/m^2^) using Asian criteria, with a mean BMI of 24.41 kg/m^2^ (SD = 4.42; median = 23.62). Most participants held a bachelor’s degree or diploma (73.07%). Nearly all were non-smokers (95.87%), and 73.25% were non-drinkers. Chronic diseases were reported by 18.67% of participants.

Regarding awareness and exposure, most parents had moderate air pollution awareness (58.17%), while 25.13% had low and 16.70% had high awareness levels. The mean awareness score was 11.06 (SD = 2.63; median = 11.00; range 5–20). Approximately 29.62% reported using air quality monitoring applications, and 37.16% wore face masks during pollution episodes. The majority (71.64%) demonstrated moderate health behaviors, with a mean health behavior score of 49.85 (SD = 10.39; median = 49.00; range 29–90). For physical symptoms, 75.40% reported at least one respiratory symptom, and nearly half (49.37%) had symptoms involving four to five body systems.

### Psychometric properties of the study scales

The three multi-item scales showed acceptable to excellent internal consistency in the analytic sample (air pollution awareness, 5 items, α = 0.70; health behaviour, 18 items, α = 0.86; psychological distress, 10 items, α = 0.91). The awareness and distress scales were only moderately correlated (r = 0.40, sharing 16.2% of variance), and awareness was essentially uncorrelated with health behaviour (r = 0.02). The item pool was suitable for factor analysis (Kaiser–Meyer–Olkin = 0.87; Bartlett’s test p < 0.001). Exploratory factor analysis of the 15 awareness and distress items yielded two factors explaining 55.8% of the variance, on which all ten distress items loaded on one factor and the awareness items measuring attention to air-quality reports, protective behaviour and community-level concern loaded on the other; the two factors were only weakly correlated (φ = 0.19). Discriminant validity was supported by both the Fornell–Larcker criterion (square root of the average variance extracted: awareness 0.68, distress 0.76, each exceeding their inter-construct correlation of 0.40) and the HTMT ratio (0.49, below the 0.85 threshold). Awareness and distress therefore behaved as distinguishable constructs rather than as alternative indicators of a single underlying dimension (S1 Appendix).

### Prevalence of psychological distress

Table 2 presents the prevalence of psychological distress. The overall prevalence of moderate-to-high psychological distress was 61.94% (95% CI: 57.87–65.85), with 50.81% classified as moderate and 11.13% as high distress. The mean psychological distress score was 25.73 (SD = 7.63; median = 25.00; range 10–48). In addition, the most frequently reported concerns at the level of frequently or more were stress upon seeing or hearing about high pollution levels (57.81%), worry about the impact on family health (56.19%), and stress about outdoor activities during high pollution days (54.94%). Notably, items reflecting helplessness about poor air quality (34.47%) and concern about adequacy of protective measures (35.91%) were also reported by more than one-third of participants.

**Table 2:** Prevalence of psychological distress related to air pollution (n = 557).

Psychological distress	Number	Percentage	95% CI
**Psychological distress level**			
Low (<23 scores)	212	38.06	34.08-42.20
Moderate (23–36 scores)	283	50.81	46.67-54.93
High (≥37 scores)	62	11.13	8.77-14.01
Moderate-to-high distress (≥23 scores)	345	61.94	57.87-65.85
Mean (SD)	25.73 (7.63)		
Median (Min:Max)	25.00 (10:48)		

Note: CI, confidence interval; SD, standard deviation; Min, minimum; Max, maximum.

### Factors associated with psychological distress among the parents of urban schoolchildren

Table 3 presents the bivariate analysis examining factors associated with moderate-to-high psychological distress. Among personal characteristics, gender showed borderline significance (overall p = 0.094), with females having 1.52 times higher odds of psychological distress compared to males (95% CI: 0.93–2.46). Age, BMI, education, healthcare coverage, smoking, drinking, and chronic disease were not significantly associated with the outcome (all p > 0.25).

**Table 3:** Factors associated with psychological distress: Simple logistic regression analysis (n = 557).

Factors	Number	% Psychological distress	COR	95% CI	p-value
**Personal characteristics**					
**Gender**					0.094
Male	77	53.25	Ref.		
Female	480	63.33	1.52	0.93-2.46	
**Age (years)**					0.250
≥46	115	56.52	Ref.		
36-45	315	61.90	1.25	0.81-1.93	
<36	127	66.93	1.56	0.92-2.62	
**BMI (kg/m²)**					0.292
Normal (18.5–22.9)	210	64.29	Ref.		
Underweight (<18.5)	31	70.97	1.36	0.59-3.10	
Overweight (23.0–24.9)	108	63.89	0.98	0.61-1.59	
Obese (≥25.0)	208	57.21	0.74	0.50-1.10	
**Education level**					0.810
Below bachelor’s degree	85	58.82	Ref.		
Bachelor’s/Diploma	407	62.41	1.16	0.72-1.87	
Above bachelor’s degree	65	63.08	1.20	0.62-2.32	
**Healthcare coverage**					0.316
Universal Health Coverage	173	57.80	Ref.		
Private Insurance/SS	237	62.45	1.21	0.81-1.81	
Civil Servant Scheme	147	65.99	1.42	0.90-2.23	
**Smoking status**					0.585
Non-smoker	534	62.17	Ref.		
Ever/Current smoker	23	56.52	0.79	0.34-1.84	
**Alcohol consumption**					0.346
Non-drinker	408	62.25	Ref.		
Former drinker	46	69.57	1.39	0.72-2.68	
Current drinker	103	57.28	0.81	0.52-1.26	
**Chronic disease**					0.302
None	453	60.93	Ref.		
Present	104	66.35	1.26	0.81-1.98	
**Awareness and exposure factors**					
**Air pollution awareness level**					<0.001
Low (<10 scores)	140	42.86	Ref.		
Moderate (10–13 scores)	324	65.12	2.49	1.66-3.73	
High (≥14 scores)	93	79.57	5.19	2.84-9.51	
**Air quality app use**					<0.001
No	392	56.89	Ref.		
Yes	165	73.94	2.15	1.44-3.21	
**Face mask wearing**					0.007
No	350	57.71	Ref.		
Yes	207	69.08	1.64	1.14-2.35	
**Close smokers in household**					0.472
None	323	60.68	Ref.		
Present	234	63.68	1.14	0.80-1.61	
**Health behaviors**					
**Health behavior level**					<0.001
Inappropriate (<42 scores)	118	39.83	Ref.		
Moderate-Appropriate (≥42 scores)	439	67.88	3.19	2.10-4.86	
**Afternoon pick-up waiting time**					0.074
≤5 minutes	307	58.31	Ref.		
6-10 minutes	114	70.18	1.68	1.06-2.66	
>10 minutes	131	63.36	1.24	0.81-1.88	
**Physical symptoms**					
**Parent respiratory symptoms**					0.001
No symptoms	137	50.36	Ref.		
Present (≥1 symptom)	420	65.71	1.89	1.28-2.79	
**Number of body systems affected**					<0.001
0-1 systems	110	49.09	Ref.		
2-3 systems	172	56.98	1.37	0.85-2.22	
4-5 systems	275	70.18	2.44	1.55-3.84	

Note: COR, crude odds ratio; CI, confidence interval; Ref., reference category; BMI, body mass index.

Among awareness and exposure factors, air pollution awareness level demonstrated the strongest bivariate association (overall p < 0.001), showing a graded gradient across categories: moderate awareness (COR = 2.49, 95% CI: 1.66–3.73) and high awareness (COR = 5.19, 95% CI: 2.84–9.51) relative to low awareness. Air quality monitoring application use (COR = 2.15, 95% CI: 1.44–3.21, p < 0.001) and face mask wearing (COR = 1.64, 95% CI: 1.14–2.35, p = 0.007) were also significantly associated. For health behaviors, those with moderate-to-appropriate behavior had 3.19 times higher odds (95% CI: 2.10–4.86) of psychological distress compared to those with inappropriate behavior (p < 0.001). Among physical symptom variables, having four to five body systems affected was associated with 2.44 times higher odds (95% CI: 1.55–3.84) compared to those with zero to one system affected, and having any respiratory symptom was associated with 1.89 times higher odds (95% CI: 1.28–2.79, p = 0.001).

Variables meeting the inclusion criterion (p ≤ 0.25) from bivariate analysis were entered into the multivariable model: gender, age, awareness level, air quality app use, face mask wearing, afternoon pick-up time, health behavior level, number of body systems affected, and chronic disease. After backward elimination, four factors remained in the final model (Table 4). The final model showed adequate fit by the Hosmer-Lemeshow test (χ²(8) = 3.41, p = 0.906) and acceptable discriminatory ability (AUC = 0.720), with an overall correct classification rate of 69.30%.

**Table 4:** Factors associated with psychological distress: Multiple logistic regression analysis (n = 557).

Factors	Number	% Psychological distress	COR	AOR	95% CI	p-value
**Awareness level**						<0.001
Low (<10 scores)	140	42.86	Ref.	Ref.		
Moderate (10–13)	324	65.12	2.49	2.41	1.56-3.72	
High (≥14 scores)	93	79.57	5.19	4.39	2.33-8.30	
**Air quality app use**						0.008
No	392	56.89	Ref.	Ref.		
Yes	165	73.94	2.15	1.79	1.17-2.74	
**Health behavior level**						<0.001
Inappropriate (<42)	118	39.83	Ref.	Ref.		
Moderate-Appropriate (≥42)	439	67.88	3.19	3.36	2.14-5.26	
**Body systems affected**						0.005
0-1 systems	110	49.09	Ref.	Ref.		
2-3 systems	172	56.98	1.37	1.31	0.78-2.19	
4-5 systems	275	70.18	2.44	2.16	1.32-3.53	

Note: COR, crude odds ratio; AOR, adjusted odds ratio; CI, confidence interval; Ref., reference category. Model fit: Hosmer–Lemeshow goodness-of-fit test χ²(8) = 3.41, p = 0.906; area under the ROC curve = 0.720; overall correct classification 69.30%.

Air pollution awareness level showed the strongest association with psychological distress, following a statistically significant graded pattern across awareness categories. Compared to parents with low awareness, those with moderate awareness had 2.41 times higher odds (AOR = 2.41, 95% CI: 1.56–3.72), and those with high awareness had 4.39 times higher odds (AOR = 4.39, 95% CI: 2.33–8.30) of moderate-to-high psychological distress (overall p < 0.001). Parents who used air quality monitoring applications had 1.79 times higher odds of distress (AOR = 1.79, 95% CI: 1.17–2.74, p = 0.008).

Health behavior level showed a notably strong positive association with psychological distress. Compared to those with inappropriate health behaviors, parents with moderate-to-appropriate health behaviors had 3.36 times higher odds of psychological distress (AOR = 3.36, 95% CI: 2.14–5.26, p < 0.001). The number of physical symptom body systems affected was also significantly associated with distress, with those experiencing symptoms in four to five body systems having 2.16 times higher odds (AOR = 2.16, 95% CI: 1.32–3.53, p = 0.002) compared to those with zero to one system affected (overall p = 0.005).

## Discussion

This study revealed a high prevalence of psychological distress related to air pollution among parents of urban schoolchildren in Khon Kaen, with nearly two-thirds (61.94%) reporting moderate-to-high distress levels. The most striking finding was an awareness–distress pattern, wherein higher air pollution awareness showed the strongest association with psychological distress, following a graded gradient that persisted after adjustment for physical symptoms, health behaviours, and other covariates. Because the data are cross-sectional, this pattern is described as an association rather than as evidence that awareness causes distress.

The prevalence of moderate-to-high psychological distress This prevalence is broadly consistent with the wider Asia-Pacific evidence base on air pollution and mental health. A systematic review and meta-analysis of epidemiological studies, predominantly from Asian settings, reported significant associations between ambient air pollution and psychological stress and anxiety disorders [19]; regional studies from China and India have similarly linked particulate exposure to elevated depression and anxiety symptoms. Within Thailand, prior work in Khon Kaen Province documented PM_2.5_ bound heavy-metal exposure and associated health risks, and a companion school-based study in the same population reported a high burden of respiratory symptoms among schoolchildren. Beyond the Asia-Pacific region, a cross-sectional study of adults in Ireland found significant associations between long-term PM_2.5_ exposure and both depression and anxiety symptoms, even at relatively low concentrations [12]. An umbrella review reported that associations between air pollution and mood disorders were supported by suggestive to highly suggestive evidence globally [3]. The high prevalence observed in Khon Kaen likely reflects the combination of genuinely poor air quality during the study period (dry season), high media coverage of pollution events, and the particularly vulnerable psychological position of parents responsible for children’s well-being.

The awareness–distress paradox identified in this study, in which higher air pollution awareness was associated with 2.41 to 4.39 times higher odds of psychological distress in a graded pattern, represents the most novel and policy-relevant finding. This pattern parallels the broader eco-anxiety literature, which has documented that greater environmental awareness and concern are associated with increased psychological distress [6,8]. Consistent with this literature, environmental concern correlates positively with distress, depression, and anxiety [8]. Previous research suggests that while moderate levels of environmental concern may motivate adaptive behaviors, higher levels can overwhelm coping resources, leading to anxiety-related feelings [9]. The finding that air quality monitoring app users had significantly higher distress further supports this interpretation, suggesting that increased environmental information access, without concurrent psychological support, may heighten distress.

Because awareness and distress were measured at the same point in time, the direction of this association cannot be determined. Parents who are already distressed about air pollution may attend more closely to air-quality reports, install monitoring applications and adopt protective behaviours, so that distress drives awareness rather than the reverse; the two may also reinforce each other. The psychometric analyses indicate that awareness and distress are distinguishable constructs, which argues against the association being an artefact of overlapping item content, but they cannot establish temporal order. The pattern is therefore best interpreted as a co-occurrence of heightened environmental awareness and psychological burden within the same individuals.

The strong positive association between health behavior level and psychological distress warrants careful interpretation. Parents with moderate-to-appropriate health behaviors had more than three times the odds of psychological distress compared to those with inappropriate behaviors. Rather than suggesting that healthy behaviors cause distress, this finding likely reflects a shared underlying driver: parents who are more aware of and concerned about air pollution are simultaneously more likely to adopt protective health behaviors and to experience heightened psychological distress. This interpretation is consistent with an awareness–behavior–distress pattern described in the eco-anxiety literature, where environmental engagement and psychological burden co-occur as manifestations of heightened environmental concern [10,20]. Similar findings have been reported, indicating that individuals most engaged in environmental protection often experience the greatest psychological toll [13].

The significant association between the number of physical symptom body systems affected and psychological distress supports the established physical-mental health nexus in environmental epidemiology. Previous studies have demonstrated that air pollution-related physical symptoms serve both as direct stressors contributing to psychological burden and as tangible evidence that reinforces health-related anxiety [21,22]. The companion study by the same research group documented a high prevalence of respiratory symptoms among children in this population [15]. The present findings extend this evidence by suggesting that parents’ own physical symptoms may also be associated with greater psychological distress, creating a compounding effect whereby physical discomfort intensifies awareness-driven psychological burden.

Several variables that were significant in bivariate analysis, including gender, face mask wearing, and afternoon pick-up duration, did not remain statistically significant in the final multivariable model. The attenuation of the gender effect after adjustment suggests that the higher psychological distress observed among female parents (63.33% vs 53.25%) may reflect underlying differences in awareness levels and health behaviors. Similarly, the loss of significance for face mask wearing and afternoon pick-up time indicates that these variables may represent markers of awareness-related concern rather than independent risk factors.

The pathophysiological mechanisms linking air pollution to psychological distress are thought to involve both direct and indirect pathways. Directly, fine particulate matter can cross the blood–brain barrier and induce neuroinflammation, disrupting neurotransmitter systems involved in mood regulation [23,24]. Indirectly, awareness of environmental threats may activate stress response systems, and prolonged activation of the hypothalamic–pituitary–adrenal axis has been associated with sustained psychological distress [14]. In the present study, the observed associations between physical symptoms (reflecting direct exposure) and awareness factors (reflecting perception of threat) and psychological distress are consistent with the possibility that both direct and indirect pathways may be involved.

These findings have important implications for public health practice. First, air pollution communication strategies should incorporate mental health considerations. Increasing awareness of pollution hazards without simultaneously providing coping resources, actionable protective measures, and psychological support may be associated with greater psychological distress in urban populations. Second, integrated interventions that simultaneously address environmental literacy, health-protective behaviors, and psychological resilience are warranted [25]. Third, healthcare systems in polluted urban areas should consider screening for and managing environment-related psychological distress, particularly among parents of young children [26]. These recommendations align with the growing call for a One Health approach that recognizes the interconnection between environmental quality, physical health, and mental well-being [3].

This study has several strengths, including an adequate sample size, a systematic multi-stage random sampling method, and comprehensive measurement across multiple domains using rigorously developed and reliability-tested instruments. The examination of both awareness-mediated and symptom-mediated pathways provides a more nuanced understanding of the air pollution–psychological distress relationship than studies focusing solely on direct exposure. However, several limitations should be acknowledged. The cross-sectional design precludes causal inference, and the directionality of the awareness-distress relationship cannot be definitively established. Although the two participating schools were selected at random, the sampling frame was deliberately restricted to schools in high-traffic locations, so the findings describe parents of children attending traffic-exposed urban schools rather than all schools in Khon Kaen or in Thailand. Recruitment from only two schools also means that observations are clustered within a small number of sites; with two clusters it was not possible to model school-level random effects, and school-specific contextual differences could not be separated from the associations reported. The distress scale was developed for this study, and although it showed high internal consistency and discriminant validity from the awareness scale, it has not been validated against a clinical reference standard, and the low, moderate and high categories rest on criterion-referenced cut-points rather than diagnostic thresholds; sensitivity analyses using the continuous score and alternative cut-points nevertheless produced consistent findings (S1 Appendix). The highest health-behaviour category contained few participants (n = 40), so its estimate was imprecise and unstable, and the variable was analysed in two levels; the three-level specification produced the same substantive conclusions and is reported in S1 Appendix. Self-reported measures may be subject to recall and social desirability biases. No direct air quality measurements were taken during the study period, limiting the ability to correlate mental health outcomes with specific pollutant concentrations. Future studies should employ longitudinal designs with objective air quality monitoring and validated clinical anxiety and depression scales to further elucidate these relationships.

## Conclusions

This study identified a high prevalence of psychological distress (61.94%) related to air pollution among parents of urban schoolchildren, with an awareness–distress paradox as the central finding. Air pollution awareness showed a significant graded association with psychological distress, alongside associations with air quality monitoring app use, health behaviours, and multi-system physical symptoms. As the study is cross-sectional, these findings describe co-occurring patterns rather than causal effects. Accordingly, awareness-raising campaigns should be paired with psychological support and practical exposure-reduction guidance, protecting both the physical and psychological well-being of urban communities facing chronic air pollution exposure.

## Supporting information

S1 Appendix Supplementary psychometric and sensitivity analyses. Item wording and scoring of the awareness, health-behaviour and psychological-distress scales; scale-specific reliability; inter-scale correlations; exploratory factor analysis; discriminant validity (Fornell–Larcker and HTMT); collinearity and influence diagnostics; and sensitivity analyses using the continuous distress score, alternative cut-points, an a priori adjustment set, and alternative categorisation of health behaviour.
(DOCX)
