# Supplementary material for: The awareness-distress paradox: Traffic-related air pollution awareness and psychological distress among the parents of urban schoolchildren in a northeastern province, Thailand
Source: PLoS One. 2026 Sep 21;21(9):e0358849. doi: 10.1371/journal.pone.0358849 (PMC13592598; doi:10.1371/journal.pone.0358849)
Supplement: Supplementary file 1 [file pone.0358849.s001.docx]

**S1 Appendix**

**Supplementary psychometric and sensitivity analyses**

*The awareness–distress paradox: traffic-related air pollution awareness and psychological distress among the parents of urban schoolchildren in a northeastern province, Thailand (PONE-D-26-15641)*

**A. Scales, item content and scoring**

All three multi-item scales were administered in Thai during face-to-face interviews and scored so that higher totals denote higher levels of the construct. Item content is summarised below; full Thai wording is available from the corresponding author.

| **Scale** | **Items** | **Response scale** | **Range** | **Content** |
| --- | --- | --- | --- | --- |
| Air pollution awareness (F1–F5) | 5 | 4-point: 1 never, 2 sometimes, 3 often, 4 routinely | 5–20 | F1 concern about local air quality; F2 knowledge of health effects; F3 attention to air-quality reports; F4 personal protective behaviour; F5 community-level concern |
| Health behaviour (G1–G18) | 18 | 5-point: 1 never to 5 routinely | 18–90 | Exercise (G1–G6), nutrition (G7–G13), rest and sleep (G14–G18) |
| Psychological distress (I1–I10) | 10 | 5-point: 1 never to 5 always | 10–50 | Worry about family health; stress on hearing pollution reports; anxiety about long-term effects; unease when air quality is poor; worry about children; frustration at inability to control pollution; stress about outdoor activities; concern about adequacy of protection; tension when commuting; helplessness |

**B. Scale-specific reliability and inter-scale correlations (n = 557)**

| **Scale** | **Items** | **Cronbach's α** | **Mean (SD)** | **Observed range** |
| --- | --- | --- | --- | --- |
| Air pollution awareness | 5 | 0.70 | 11.06 (2.63) | 5–20 |
| Health behaviour | 18 | 0.86 | 49.85 (10.39) | 29–90 |
| Psychological distress | 10 | 0.91 | 25.73 (7.63) | 10–48 |

Inter-scale Pearson correlations: awareness–distress r = 0.403 (shared variance 16.2%); awareness–health behaviour r = 0.024 (p = 0.57); health behaviour–distress r = 0.232. Spearman coefficients were essentially identical (0.392, 0.033 and 0.240, respectively).

**C. Exploratory factor analysis of awareness and distress items**

Factorability was confirmed (Kaiser–Meyer–Olkin = 0.865; Bartlett's test of sphericity χ² = 9364.9, p < 0.001). Principal-axis extraction with promax (oblique) rotation was applied to the 15 awareness and distress items. Two factors with eigenvalues above unity were retained (6.41 and 2.02), explaining 55.8% of the total variance. Loadings ≥ 0.40 are shown in bold.

| **Item** | **Factor 1 (distress)** | **Factor 2 (awareness)** |
| --- | --- | --- |
| F1 Concern about local air quality | **0.460** | 0.272 |
| F2 Knowledge of health effects | **0.501** | 0.275 |
| F3 Attention to air-quality reports | 0.108 | **0.759** |
| F4 Personal protective behaviour | -0.007 | **0.776** |
| F5 Community-level concern | -0.060 | **0.735** |
| I1 Worry about family health | **0.835** | -0.088 |
| I2 Stress on hearing pollution reports | **0.863** | -0.062 |
| I3 Anxiety about long-term effects | **0.618** | 0.200 |
| I4 Unease when air quality is poor | **0.820** | 0.012 |
| I5 Worry about children's health | **0.843** | 0.030 |
| I6 Frustration at inability to control | **0.582** | -0.309 |
| I7 Stress about outdoor activities | **0.530** | -0.355 |
| I8 Concern about adequacy of protection | **0.776** | 0.051 |
| I9 Tension when commuting | **0.840** | 0.043 |
| I10 Helplessness about air quality | **0.763** | 0.154 |

All ten distress items loaded on Factor 1. The three awareness items reflecting attention to reports, protective behaviour and community concern (F3–F5) loaded cleanly on Factor 2. The two awareness items phrased in terms of concern and knowledge (F1, F2) loaded primarily on the distress factor, indicating partial content overlap; these items were nevertheless retained because the five-item scale was specified a priori. The inter-factor correlation was low (φ = 0.193).

**D. Discriminant validity**

| **Criterion** | **Value** | **Threshold / result** |
| --- | --- | --- |
| AVE, awareness (5 items) | 0.460; √AVE = 0.679 | √AVE > r (0.403): met |
| AVE, distress (10 items) | 0.576; √AVE = 0.759 | √AVE > r (0.403): met |
| Composite reliability, awareness / distress | 0.81 / 0.93 | > 0.70: met |
| HTMT ratio, awareness vs distress | 0.490 | < 0.85: met |
| HTMT using F3–F5 only | 0.230 | < 0.85: met |

Both the Fornell–Larcker criterion and the HTMT ratio supported discriminant validity between air pollution awareness and psychological distress. Restricting awareness to the three items without affective content (F3–F5) reduced the correlation with distress to r = 0.183 (α = 0.73; AVE = 0.652; HTMT = 0.230) while preserving the direction and significance of the association with distress, indicating that the observed relationship is not an artefact of shared item wording.

**E. Collinearity and influence diagnostics for the final model**

Variance inflation factors were 1.08 (awareness), 1.03 (health behaviour), 1.03 (air-quality application use) and 1.09 (number of body systems affected), indicating no meaningful collinearity among the model covariates. The maximum Cook's distance was 0.028 and the maximum leverage 0.054; 21 observations (3.8%) exceeded the conventional 4/n threshold. Excluding these observations left the awareness association unchanged in direction and significance. In the three-level specification, exclusion of influential observations produced quasi-separation in the highest health-behaviour category, confirming that this single estimate was unstable; this was the basis for analysing health behaviour in two levels.

**F. Sensitivity analyses**

**F.1** Continuous distress score (linear regression). Using the untransformed 10–50 distress score, the same pattern was observed: moderate awareness b = +2.72 (95% CI +1.34 to +4.09), high awareness b = +6.59 (+4.75 to +8.42), moderate-to-appropriate health behaviour and application use also positively associated; model R² = 0.215.

**F.2** Alternative cut-points for the distress outcome. The graded awareness association was robust to the choice of threshold:

| **Cut-point for the outcome** | **n with outcome** | **High vs low awareness, AOR (95% CI)** |
| --- | --- | --- |
| ≥ 23 (primary analysis) | 345 | 4.39 (2.33–8.30) |
| ≥ 20 | 474 | 5.37 (1.80–16.04) |
| ≥ 26 | 263 | 4.21 (2.31–7.68) |
| ≥ 30 | 153 | 4.88 (2.55–9.32) |

**F.3** A priori adjustment set instead of backward elimination. Entering awareness, application use, health behaviour, number of body systems affected, sex and age group simultaneously and retaining all of them produced essentially unchanged estimates (moderate awareness AOR 2.44, 95% CI 1.58–3.79; high awareness AOR 4.20, 95% CI 2.20–8.02), with sex (AOR 0.96) and age group not significantly associated.

**F.4** Categorisation of health behaviour. The highest health-behaviour category contained only 40 participants, of whom 33 (82.5%) reported moderate-to-high distress. Its estimate was correspondingly imprecise, with a confidence interval spanning a 6.7-fold range, and it collapsed into quasi-separation when influential observations were trimmed. Health behaviour was therefore analysed in two levels in the final model. The table below shows that this decision does not affect the study's conclusions: the estimates for air pollution awareness, application use and symptom burden are essentially unchanged under either specification.

| **Variable** | **Three-level model**  **AOR (95% CI)** | **Two-level model (final)**  **AOR (95% CI)** |
| --- | --- | --- |
| Awareness, moderate vs low | 2.47 (1.60–3.83) | 2.41 (1.56–3.72) |
| Awareness, high vs low | 4.38 (2.31–8.31) | 4.39 (2.33–8.30) |
| Air-quality application use | 1.72 (1.12–2.65) | 1.79 (1.17–2.74) |
| Health behaviour, moderate vs inappropriate | 3.13 (1.99–4.92) | - |
| Health behaviour, appropriate vs inappropriate | 9.45 (3.66–24.45) | - |
| Health behaviour, moderate-to-appropriate vs inappropriate | - | 3.36 (2.14–5.26) |
| Body systems affected, 2–3 vs 0–1 | 1.33 (0.79–2.25) | 1.31 (0.78–2.19) |
| Body systems affected, 4–5 vs 0–1 | 2.35 (1.42–3.88) | 2.16 (1.32–3.53) |

The highest health-behaviour category contained 40 participants, of whom 33 (82.5%) reported moderate-to-high distress, yielding a wide confidence interval in the three-level specification. Health behaviour was therefore analysed in two levels in the final model reported in the main text, while the three-level distribution is retained in Table 1 for descriptive completeness.
